# Optimization of supercritical-CO_2_ extraction and pharmacokinetics in SD rats of alkaloids form *Sophora moorcroftiana* seed

**DOI:** 10.1038/s41598-022-07278-1

**Published:** 2022-02-28

**Authors:** Chunhui Hu, Xuehui Gan, Qiangqiang Jia, Pan Gao, Tao Du, Fabin Zhang

**Affiliations:** 1 Medical College, Qinghai University, 251 Ningda Road, Xining, 810001 Qinghai People’s Republic of China; 2 State Key Laboratory of Plateau Ecology and Agriculture, Qinghai University, Xining, 810001 Qinghai People’s Republic of China; 3 Department of Hepatopancreatobiliary Surgery Affiliated Hospital of Qinghai University, Xining, 810001 Qinghai People’s Republic of China

**Keywords:** Drug discovery, Pharmacology, Pharmacokinetics

## Abstract

The total alkaloids extracted from the seeds of *Sophora moorcroftiana* (TAs-SM) have the potential to treat alveolar echinococcosis, a disease included by the WHO in a list of 17 key neglected diseases world-wide. The aims of the current study were first to develop a supercritical fluid extraction (SFE) method for optimizing TAs-SM extraction, and second, to develop an optimized method for evaluating TAs-SM pharmacokinetics in vivo. The Box–Behnken response surface method was used to optimize the extraction process, and ultra-high liquid chromatography coupled with high resolution electrospray mass spectrometry (UPLC-HR-ESI-MS) was used to determine the pharmacokinetics of TAs-SM in SD rats. The results indicated the following optimal SFE extraction conditions: pressure = 31 MPa, temperature = 70 °C, time = 162.18 min. With these parameters, total alkaloids could be extracted from each gram of *S*.* moorcroftiana*, with the total content being 68.88 μg. The linear range of UPLC-HR-ESI-MS is 0.78–200.00 ng/ml, R^2^ > 0.99, and the sample recovery is 99–113%. The precision, accuracy, selectivity and stability of the method meet the requirements of US FDA guidelines. To our knowledge this study is the first to establish an SFE method for extracting TAs-SM and the first to employ UPLC-HR-ESI-MS for measuring TAs-SM in rats. These findings provide important contributions for using TAs-SM in further drug development and clinical applications.

## Introduction

*Sophora moorcroftiana* (SM) is a plant unique to the Qinghai–Tibet Plateau whose seeds are used in medicine^1–3^. It has anti-inflammatory, detoxification and other beneficial effects, and as such is an extremely valuable medicinal resource. At present, there are 13 kinds of active ingredients in SM, including alkaloids, flavonoids, esters, steroids, fatty acids and fatty acid esters^4,5^. In recent years, with the in-depth study of alkaloid biological activities^6,7^, it has been found that alkaloids have a variety of biological effects, including anti-bacterial, anti-inflammatory, anti-tumor, anti-liver injury and immune enhancement effects^8–15^.

Echinococcosis is one of the ten diseases with the heaviest economic burden in the world and has been listed by the WHO as one of 17 key neglected diseases to be controlled or eliminated by 2050^16^. Our previous study found that the total alkaloids (TAs; including matrine, oxymatrine, sophocarpine and sophoridine) extracted from the seeds of SM can be used to effectively inhibit alveolar echinococcosis, and the TAs combined with the commercial drug albendazole can inhibit the growth of lesions by improving liver and immune function^17^. Other researchers have found that TAs combined with BCG can be used to treat secondary echinococcosis in mice by increasing the number of CD4^+^ T cell subsets, preventing the induced expression of PD-1 on the surface of T cells, and promoting cellular immune function^14,18–20^.

The choice of extraction process is of great significance for maximizing the pharmacological effects of traditional Chinese medicines (TCMs). Typical extraction methods for alkaloids are acid water extraction^21,22^. Most of these methods require organic solvents, have low safety factors and have the potential to leave organic solvent residues, creating problems that make these processes difficult to industrialize^22–24^. As an alternative, the supercritical fluid extraction (SFE) technique is a method that uses supercritical fluid as an extractant to separate extracts from a matrix. This method has both liquid solubilizing capacity and good gas flow and transport efficiency. CO_2_ is nontoxic, stable in nature, and easy to separate. Additionally, the CO_2_ critical temperature and pressure are low and easy to industrialize. These advantages make CO_2_ one of the most used supercritical fluids and several TCM components have been successfully extracted using supercritical extraction techniques, including alkaloids, flavonoids, quinones, as well as volatile oil compounds^23,25,26^. In the current study we employ a supercritical CO_2_ fluid extraction technique to extract TAs from SM (TAs-SM), overcoming the shortcomings of organic solvent residue and difficult industrial production inherent in other extraction techniques.

Pharmacokinetics is a “bridge discipline” in modernizing the research chain of TCM. In a previous study, our group established a high-performance liquid chromatography (HPLC) assay for TAs-SM^27^. However, the in vivo drug concentrations of serum have been found to be far below the detection limit of HPLC due to various factors including drug absorption, drug metabolism, and the complex physiological environment. To our knowledge, there are no related reports on the in vivo pharmacokinetics of TAs-SM. In this study, a method using ultra-high liquid chromatography coupled with high resolution electrospray mass spectrometry (UPLC-HR-ESI-MS) was developed to analyze matrine, oxymatrine, sophocarpine and sophoridine together in rat plasma. Our findings provide a pharmacokinetic method and novel data to characterize the effectiveness and toxic side effects of TAs-SM, which can motivate subsequent related pharmaceutics studies.

## Material and methods

### Materials and reagents

For SFE, we used an SFT-100 supercritical fluid extractor (Supercritical Fluid Technologies, USA). We also used a LG-02 high speed traditional Chinese medicine pulverizer (Ruian Baixin pharmaceutical equipment factory), Q-Orbitrap mass spectrometer (Thermofisher Scientific, USA), and an Accucore aQ UHPLC column (150 mm × 2.1 mm × 2.6 μm).

**Figure 1:**
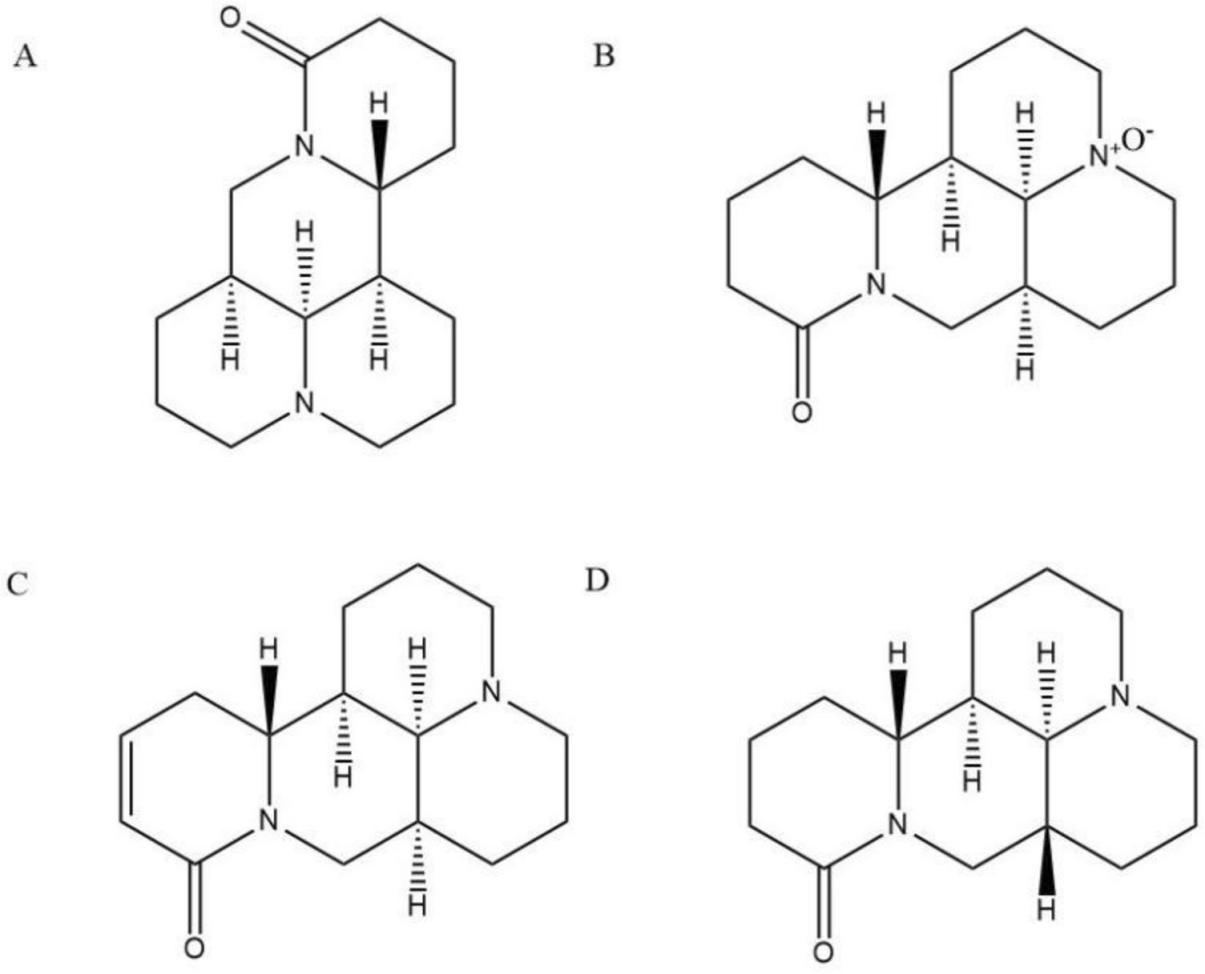
Structural formula of (**A**) Matrine, (**B**) Oxymatrine, (**C**) Sophocarpine, (**D**) Sophoridine.

Key reagents are as follows: oxymatrine, sophocarpine, sophoridine and matrine standard (Tianjin Alta Technology Co., Ltd., batch number 16837-52-8, 6483-15-4, 6882-68-4, 519-02-8) (Fig. 1), mebendazole reference (Tianjin Alta Technology Co., Ltd., batch number 31431-39-7), methyl alcohol as chromatographic purity, CO_2_ (purity 99.9%, Xining Xingwei Gas Industry Co., Ltd.), isoflurane (Shenzhen Reward Life Technology Co., Ltd.). *Sophora moorcroftiana* seeds were purchased from Linzhi, Tibet, and identified as the dried seeds of *Sophora moorcroftiana* by Associate Professor Yang Shibin of Medical College of Qinghai University.

### Experimental animals

Ten male SD rats of SPF grade, weighing 200–220 g, were provided by Shanghai Jiesijie Experimental Animal Co., Ltd. with license number SCXK (Shanghai) 2018-0004 and certificate number 20180004026773. They were used in experiments after one week of adaptive feeding. All animal experiments were reported to and have been approved by the medical ethics committee of the Medical College of Qinghai University.

### SFE-CO_2_ method

The effects of varying parameter values were evaluated for each parameter separately in a series of experiments. The factors of SFE-CO_2_ included pressure, temperature, time, alkalization time, the carrier ethanol concentration, and crushing time of the sample to be extracted. We used 10.00 g of SM seeds powder, and extracted alkaloids with the following parameter values: pressure = 17, 24, or 31 MPa; temperature = 50, 60, or 70℃; extraction time = 60, 120, or 180 min; alkalization time = 3, 5, or 8 min; concentration of ethanol (as the entrainer) = 20, 50, or 80%, and the crushing time of the sample to be extracted = 1, 2, or 3 min^27^. The extraction was repeated three times and the average value was taken.

### Experimental design for Box–Behnken response surface method

Following the results of the single factor experiments, pressure, temperature and extraction time were chosen as the key SFE-CO_2_ parameters, with the parameter values that had optimized extraction in the experiments. The subsequent experimental design was a response surface experiment with three factors and three levels (Table 1). After calculating the fitting equation, the optimum conditions for the fluid extraction of TAs-SM through pole picking were applied to the SFE-CO_2_ extraction method to optimize the process.

**Table 1:** Factors studied and their levels.

Factors	Levels		
	A: Extraction pressure (MPa)	B: Extraction temperature (℃)	C: Extraction time (min)
− 1	− 1	17	50
0	0	24	60
1	1	31	70

### UPLC-HR-ESI-MS analysis

#### Chromatographic conditions

A Dionex Ultimate 3000 RSLC system (Thermofisher) combined with an Accucore aQ C_18_ column (150 mm × 2.1 mm, 2.6 μm, Thermo fisher) was used in the sample separation process, and the separation speed was set at 0.3 μL/min for all the gradients. 1 μL of injection volume and a constant temperature of (25 ± 1) °C were adopted in the column. The eluents were A, water with 0.1% formic acid (v/v) and B, methanol, and the gradient program was as follows: 0–6 min, 8–40% B; 6–8 min, 40–100% B; 8–10 min, 100% B; 10–11 min, 100–8% B; 11–13 min, 8% B.

#### Mass spectrometry conditions

Heated electro-spray ionization (HESI) was paired with a Q-Orbitrap MS in the MS analysis. The flow rate of auxiliary, sheath and sweep gas were set at 35, 10 and 1 (arbitrary unit), respectively. A full MS-ddMS^2^ mode was used to perform the analysis. The damping gas was in the C-trap and nitrogen was used to stabilize the spraying. Temperatures of 350 °C and 320 °C were set and kept for the auxiliary gas heater and capillary. Under negative mode, 3.0 kV was adopted for the spray voltage, 60 V was used for the S-lens RF level, 50 ms was used for the maximum injection time, and 3.0 e^6^ was set as the automatic gain control target. Full MS-ddMS^2^ scan ranged from 150.0000 to 800.0000 m/z. Precise molecular weight [M-H]^+^ was used for qualitative analysis, the corresponding peak area was used for quantitative analysis, and MS^2^ fragments were used for further qualitative analysis.

### Preparation of reserve sample, internal standard (IS) sample, standard sample and quality control sample

#### Stock samples

The stock sample (gavage fluid) was the TA solution extracted by SFE-CO_2_, and the administration dose was 3 mg/kg (matrine 63.69 μg/kg, oxymatrine 5.10 μg/kg, sophorine 11.07 μg/kg, and sophoridine 0.30 μg/kg) which was stored at 4 °C.

#### IS samples

An accurate measurement of 100 μg/mL mebendazole solution was dissolved in a 50.00 mL volumetric flask with chromatographic grade methanol to form a total of 200 ng/mL, which was stored at 4 °C.

#### Standard samples

Matrine (1.03 mg), oxymatrine (1.12 mg), sophorine (0.94 mg), and sophordine (0.96 mg) were accurately weighed and co-placed in a 10.00 mL volumetric flask. Methanol of chromatographic grade was added to dissolve the volume and obtain the respective alkaloid contents of 103, 112, 94 and 96 μg/mL in the mixture. Following this, 100 μL of the mixed solution was added to chromatography-grade methanol in a 10.00 mL volumetric flask to obtain the standard mixed sample with the alkaloid contents of 1.03, 1.12, 0.94 and 0.96 μg/mL, respectively.

#### Quality control (QC) samples

An appropriate amount of the stock samples solution was accurately absorbed and added into blank plasma, and then diluted into a standard plasma QC sample working solution with a TA of 1.56, 25.00 or 100.00 ng/mL and with low, medium or high concentrations.

### Method validation

The UPLC-HR-ESI-MS method was validated according to the US Food and Drug Administration (FDA) guideline for bioanalytical method validation^28^ and included determination of selectivity, linearity, lower limit of quantitation (LLOQ), accuracy, precision, matrix effect, recovery and stability.

#### Selectivity

The selectivity of the method was investigated by comparing the chromatograms of rat blank plasma samples, blank plasma samples + IS, blank plasma samples + mixed standard + IS, and plasma samples 1 h after intragastric administration.

#### Linearity and LLOQ

In order to calculate standard curves, we took the standard sample and added blank plasma, diluted by the gradient, then added 100μL 0.2 μg/mL of the IS mebendazole. In accordance with the treatment method for blood samples, the standard curve concentrations of working solutions for the four alkaloids were obtained as follows: matrine = 206.00, 103.00, 51.50, 25.75, 12.88, 6.44, 3.22, 1.61, and 0.80 ng/mL; oxymatrine = 224.00, 112.00, 56.00, 28.00, 14.00, 7.00, 3.50, 1.75, and 0.87 ng/mL; sophorine = 188.00, 94.00, 47.00, 23.50, 11.75, 5.88, 2.94, 1.47, and 0.73 ng/mL; and sophoridine = 192.00, 96.00, 48.00, 24.00, 12.00, 6.00, 3.00, 1.50, and 0.75 ng/mL. The assay was performed according to the UPLC-HR-ESI-MS conditions (see "UPLC-HR-ESI-MS analysis" section) and depending on the concentrations of the ingredients to be tested. The linear regression equation was evaluated by taking the ratio of the peak area ratio of each analyte to the IS as the ordinate, and using the weighted least square method. The lower limit of detection (LLOD) and the LLOQ were determined as the concentrations at signal-to-noise ratios of 3 and 10, respectively.

#### Accuracy and precision

Five standard plasma QC samples with low, medium and high drug concentrations were taken, and each concentration was measured in parallel five times, three times a day for three consecutive days. The concentrations of matrine, oxymatrine, sophocarpine and sophoridine in the samples were calculated, and the intra-day and inter-day precision and accuracy of the method were evaluated.

#### Extraction recovery and matrix effects

QC samples with low, medium and high concentrations (n = 5) were taken to analyze the chromatographic peak areas of matrine, oxymatrine, sophorine, sophoridine and IS compounds, and the extraction recoveries were determined.

Matrix effects were evaluated by comparing the peak area of QC samples added to blank plasma and the same concentration of pure standard solution (n = 5).

#### Stability

QC samples (n = 5) with low, medium and high concentrations of standard plasma were prepared. The sample concentrations were determined at 25 °C for 24 h, 4 °C for 24 h and—80 °C for 15 d, with repeated freezing and thawing three times.

### Comparison of systemic pharmacokinetics in SD rats

A cross-over design was employed with male SD rats (n = 10) to evaluate in vivo pharmacokinetics. The rats were subjected to fasting one night before each administration and were fed 4 h after administration. TAs-SM were given by gavage (3 mg/kg). Blood was collected under isoflurane anesthesia at a flow rate of 2.0 mL/min. At 0.25, 0.5, 1, 2, 2.5, 3, 4, 6, 8, 12 and 24 h after administration, 200–400 μL blood was collected from the orbital venous plexus and centrifuged at 13,200 rpm for 5 min. The upper plasma was stored at − 80 °C for testing.

After plasma sample melting, we took 100 μL, added an equal volume of mass spectrometry grade methanol, vortexed the sample for 1 min, centrifuged the sample at 13,000 rpm for 5 min. We then took 100 μL of the supernatant, added 100 μL of 200 ng/mL mebendazole IS solution, vortexed the sample for 1 min, and finally centrifuged the sample at 13,000 rpm for 5 min. The resulting sample was injected by UPLC-HR-ESI–MS with an injection volume of 1 μL. The pharmacokinetic study of SD rats was performed according to the standards recommended by the "Guidelines for the Care and Use of Laboratory Animals" (Animal Laboratory Resources Institute, 1995) and was approved by the Institutional Animal Care and Use Committee of Qinghai University. All animal experimental methods reported in this study were in accordance with Animal Research: Reporting of In Vivo Experiments (ARRIVE) guidelines 2.0^29^.

### Data statistics and analysis

SPSS 22.0 software was used for statistical analysis. The measurement data were expressed as mean ± standard deviation. The comparison of multiple samples was analyzed by ANOVA, with a significance level of *P* < 0.05. Figures were created using GraphPad Prism 8.3.0 software. Design Expert 8.0 software was used to fit the experimental data for the Box-Behnken modeling of the response surface. The pharmacokinetic parameters were assessed by non-compartment model analysis using Das 3.2.8.

## Results and discussion

### Optimization of SFE-CO_2_

#### Single factor experiments

SFE-CO_2_ single factor screening results showed that the extraction amount increased with the increase of extraction pressure (F = 29.490, *P* < 0.001), with a smaller increase in TAs content from 17 to 24 MPa, and a larger increase from 24 to 31 MPa.

Increasing extraction temperature also significantly increased TAs extraction (F = 6.395, *P* = 0.033). When the extraction temperature increased from 50 to 70 °C, the content of TA increased. The increase in TAs extracted was larger when the temperature increased from 50 to 60 °C than when it further increased from 60 to 70 °C.

Finally, increased extraction time was associated with increased TAs content (F = 22.030, *P* = 0.002), and this was most marked from 60 to 120 min, with a smaller change from 120 to 180 min.

**Figure 2:**
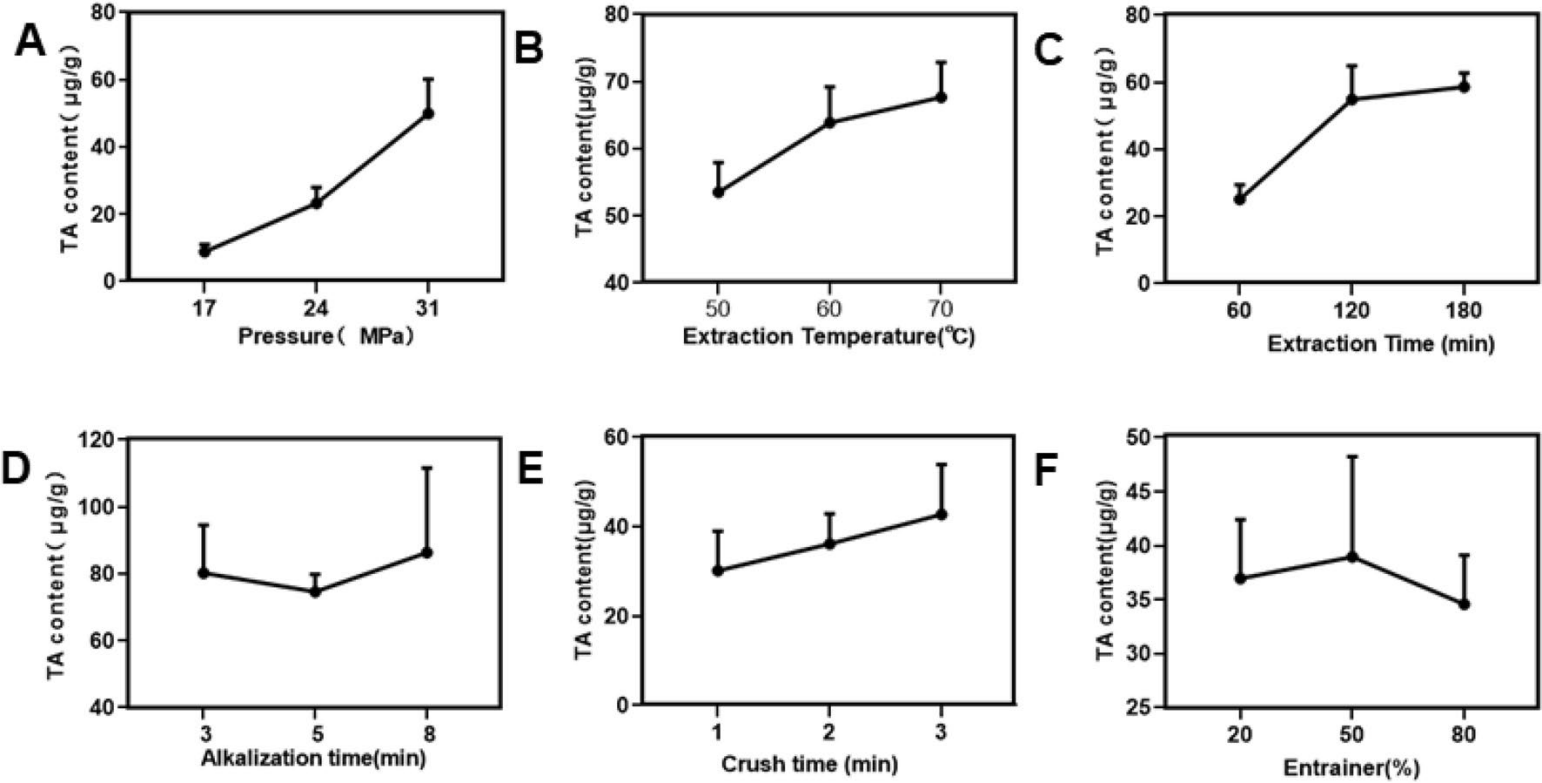
Single factor analysis diagram of Supercritical CO_2_ Fluid Extraction. (**A**) extraction pressure, (**B**) extraction temperature, (**C**) extraction time, (**D**) alkalization time, (**E**) crushing time, (**F**) content of entrainment agent.

Other experiments showed that there was no significant effect on extraction of alkalization time, entrainer concentration or sample crushing time (*P* > 0.05). The experimental results are shown in Table 2 and Fig. 2.

**Table 2:** Single factor analysis table of Supercritical CO_2_ Fluid Extraction process.

Experimental conditions	N	Mean ± SD	F-value	*P*-value
**Extraction pressure (MPa)**				
17	3	8.85 ± 2.14	29.490	< 0.001
24	3	23.24 ± 4.75		
31	3	49.89 ± 10.26		
**Extraction temperature (℃)**				
50	3	53.50 ± 4.41	6.395	0.033
60	3	63.86 ± 5.36		
70	3	67.65 ± 5.23		
**Extraction time (min)**				
60	3	25.14 ± 4.34	22.030	0.002
120	3	54.89 ± 10.06		
180	3	58.62 ± 4.18		
**Alkalization time (min)**				
3	3	80.23 ± 14.27	0.357	0.714
5	3	74.63 ± 5.20		
8	3	86.35 ± 25.21		
**Crushing time (min)**				
1	3	30.19 ± 8.83	1.447	0.307
2	3	36.18 ± 6.67		
3	3	42.79 ± 11.16		
**Carrier concentration (%)**				
20	3	36.95 ± 5.45	0.318	0.739
50	3	38.90 ± 9.29		
80	3	34.57 ± 4.55

#### Optimization of Box–Behnken response surface

Based on the results of the single factor screening experiments, three factors were selected for inclusion in a Box–Behnken design for optimizing the number of extracted TAs: pressure, temperature and extraction time. The results of this optimization are shown in Table 3.

**Table 3:** Design and results of response surface optimization for extraction conditions (n = 5).

Run	A:Pressure (MPa)	B: Temperature (℃)	C: Extraction time (min)	TA-SMs (Mean ± SD)
1	− 1	− 1	0	42.74 ± 2.89
2	1	− 1	0	47.34 ± 1.76
3	− 1	1	0	45.16 ± 1.50
4	1	1	0	57.91 ± 1.98
5	− 1	0	− 1	20.15 ± 0.79
6	1	0	− 1	27.04 ± 1.26
7	− 1	0	1	46.62 ± 2.02
8	1	0	1	61.30 ± 2.19
9	0	− 1	− 1	26.34 ± 1.99
10	0	− 1	− 1	23.12 ± 0.90
11	0	1	1	42.30 ± 1.52
12	0	1	1	62.12 ± 1.84
13	0	0	0	53.12 ± 1.98
14	0	0	0	53.25 ± 2.07
15	0	0	0	53.74 ± 1.45
16	0	0	0	52.36 ± 1.95
17	0	0	0	55.32 ± 0.99

Design expert 8.0 software was used to conduct a multiple regression fitting on the data, and the following quadratic regression equation with TAs-SM content as the index was obtained: Y = 53.55 + 4.87A + 3.7B + 14.46C + 2.04AB + 1.95AC + 5.76BC − 2.48A^2^ − 2.79B^2^ − 12.30C^2^ (A = pressure, B = temperature and C = extraction time). There was a statistical difference in the established model (P < 0.001), and the error was not statistically significant (R^2^ = 0.9955). The adjusted coefficient of determination was R^2^_adj_ = 0.9898. The model can account for 98.98% response variability. The results are shown in Table 4.

**Table 4:** ANOVA for reduced quadratic model.

Source	Sum of squares	df	Mean square	F-value	*P*-value
Model	2869.41	9	318.82	173.42	< 0.001
A-pressure	189.35	1	189.35	102.99	< 0.001
B-temperature	109.45	1	109.45	59.53	0.001
C-time	1673.02	1	1673.02	910.00	< 0.001
AB	16.61	1	16.61	9.03	0.198
AC	155.17	1	15.17	8.25	0.024
BC	132.71	1	132.71	72.18	< 0.001
A^2^	25.89	1	25.89	14.08	0.007
B^2^	32.70	1	32.70	17.79	0.004
C^2^	636.70	1	636.70	346.32	< .0001
Residual	12.87	7	1.84	–	–
Lack of fit	8.02	3	2.67	2.20	0.230
Pure error	4.85	4	1.21	–	–
Cor total	2882.28	16		–	–
	R^2^ = 0.9955		R^2^_adj_ = 0.9898		

#### Interaction effects in the Box–Behnken response surface analysis

**Figure 3:**
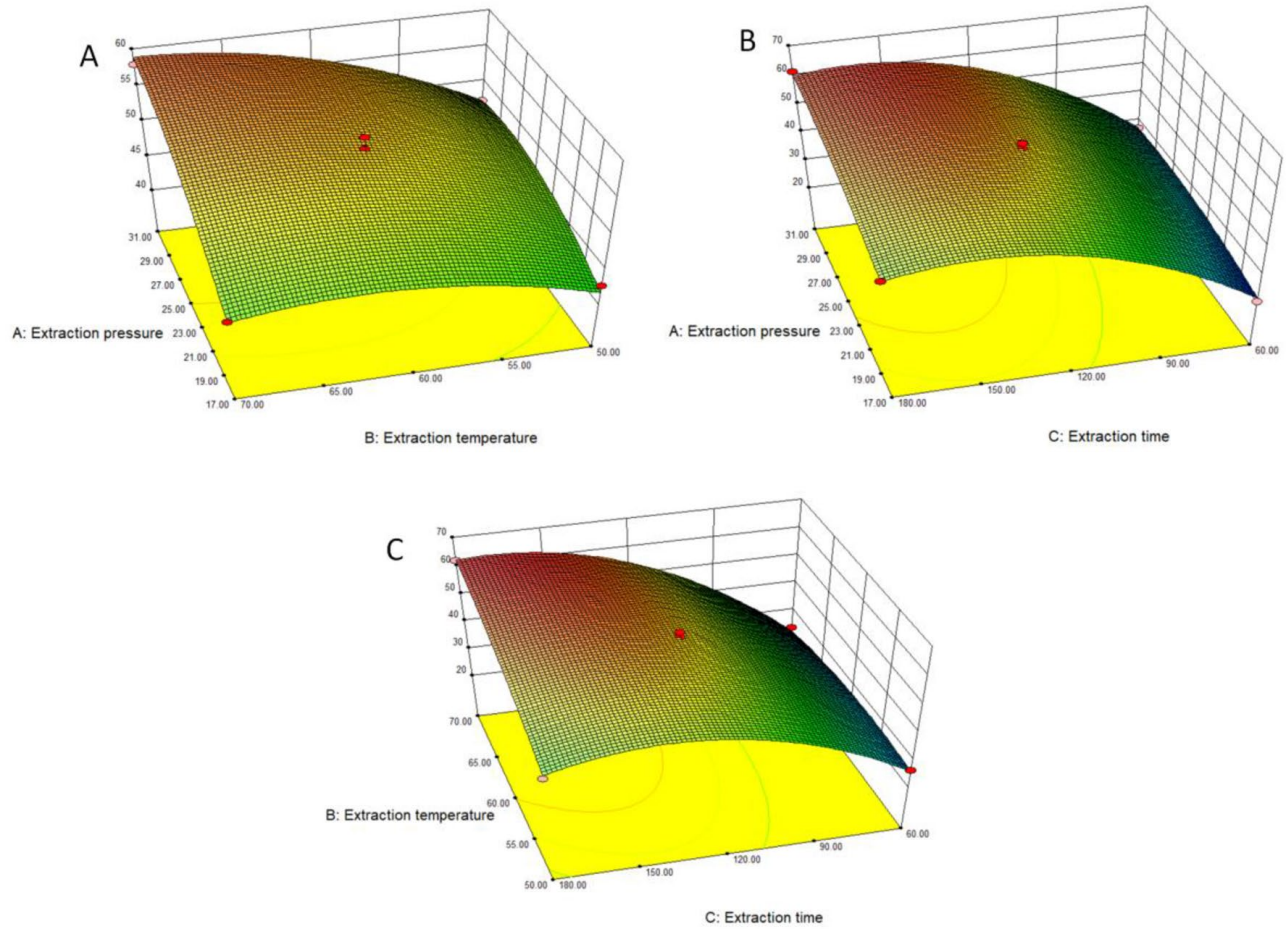
The effect of the interaction of pairs of factors on the extraction of total alkaloids. (**A**) Interaction between extraction pressure and extraction temperature, (**B**) Interaction between extraction time and extraction temperature, (**C**) Interaction between extraction time and extraction pressure.

The effects of the interaction of the three factors (pressure, temperature, and extraction time) on the TAs extraction efficiency were investigated. The more tortuous the response surface is, the more intense the color change is, indicating a stronger interaction. There was no significant interaction between extraction pressure and temperature (F = 9.03, *P* = 0.198). The slope of the response surface curve was small, and the color change trend was not evident. There was a significant interaction between extraction time and pressure (F = 8.25, *P* = 0.024). Finally, there was a significant interaction between extraction time and temperature (F = 72.18, *P* < 0.001), and the response surface had obvious slope and strong color contrast. The results are shown in Fig. 3.

#### Validation experiment

Based on the established mathematical model, we carried out parameter optimization analysis and the extremums of factors A, B and C were calculated. The results indicated that when the extraction pressure was 31 MPa, the extraction temperature was 70 °C and the extraction time was 162.18 min, the model predicted the highest TAs extraction, with a predicted value of 68.88 μg/g. The mean value of the validation experiment was 68.67% μg/g (n = 3), which is close to the model’s predicted value. The model has a small error and successful fitting. The results of the validation experiment are shown in Table 5.

**Table 5:** Model prediction verification experiment result table.

Numbering	Measured total alkaloid content (μg/g)	Average value (μg/g)	RSD (%)
1	69.02		
2	67.28	68.67	1.82
3	69.71		

In recent years, an increasing number of studies in traditional Chinese medicine have utilized SFE-CO_2_ to extract effective components^30,31^. Compared with traditional organic extraction methods, SFE has many advantageous characteristics, such as high extraction capacity, improved extraction efficiency and ease of industrialization^32^. When Hegel et al.^31^ used SFE-CO_2_ to extract alkaloids from plants, they found that higher concentrations of alkaloids were obtained by SFE-CO_2_ than by using the conventional solvent methanol. Likewise, A.R. Guedes et al., extracted *synapenium grantii hook F*. using conventional solvents and the method of SFE-CO_2_ plus ethanol; using SFE-CO_2_ improved the resulting target components by nearly 10 times compared to ethanol extraction^33^. Given this evidence that SFE-CO_2_ extraction is more efficient and more successful at guaranteeing the biological activity of the extract, we chose SFE-CO_2_ for the extraction of TAs-SM.

For the extraction of alkaloids by SFE-CO_2_, previous research suggests that pressure, temperature, extraction time, alkalization treatment time, crushing particle size, and entrainer concentration all affect the experimental results^31,32,34–36^. Thus, we included these factors in our single factor experiments. The results of the single factor experiments demonstrated that some of these factors did not affect extraction, including alkalinization treatment time, particle size, and entrainer concentration.

### Method validation

#### Selectivity

**Figure 4:**
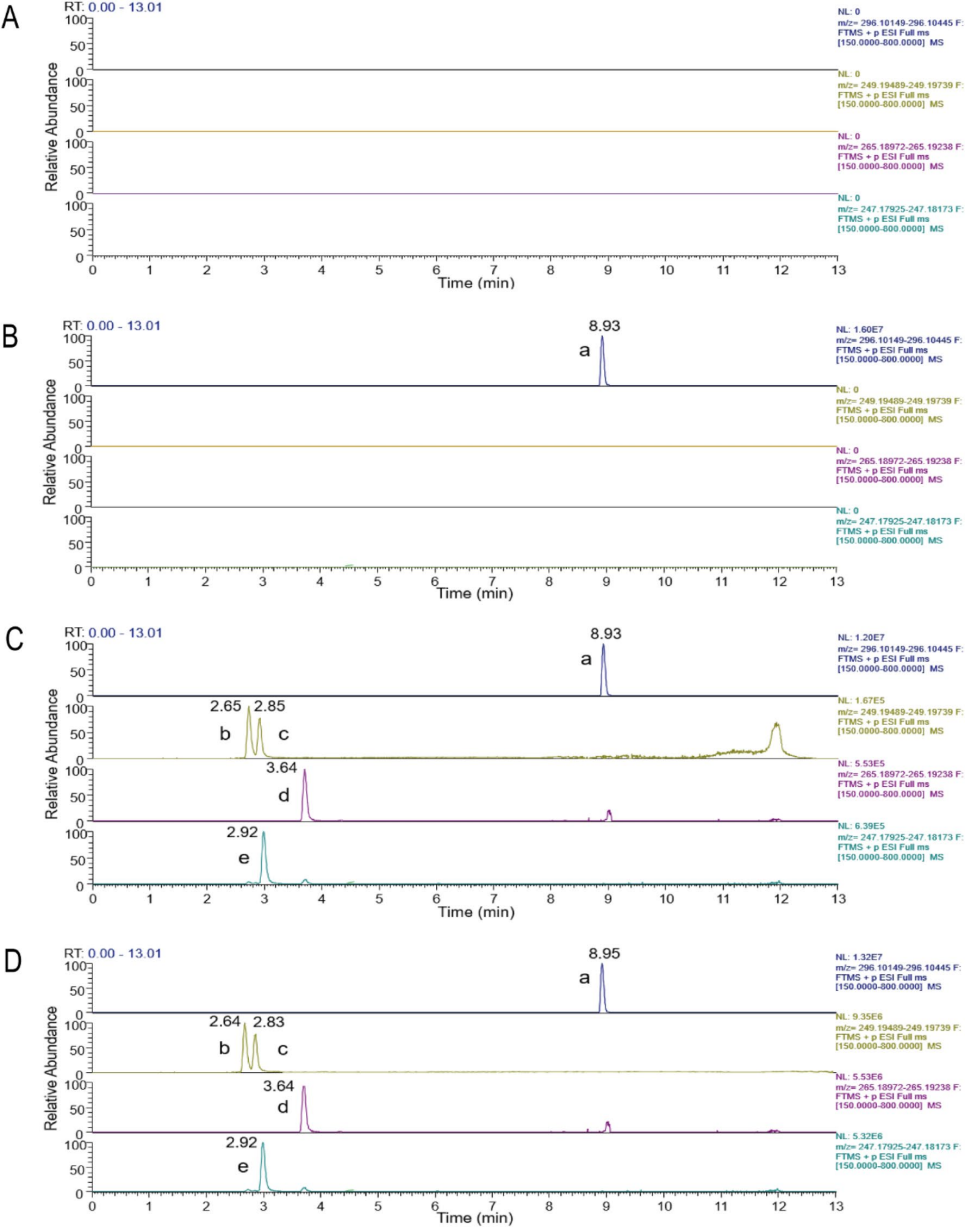
Chromatogram of (**A**) Blank plasma, (**B**) Blank plasma + Internal standard, (**C**) Blank plasma + Standard solution + Internal standard, (**D**) Rat plasma sample + Internal standard + after 1 h of Intragastric administration. (a) Mebendazole, (b) Matrine, (c) Sophoridine, (d) Oxymatrine, (e) Sophocarpine.

**Figure 5:**
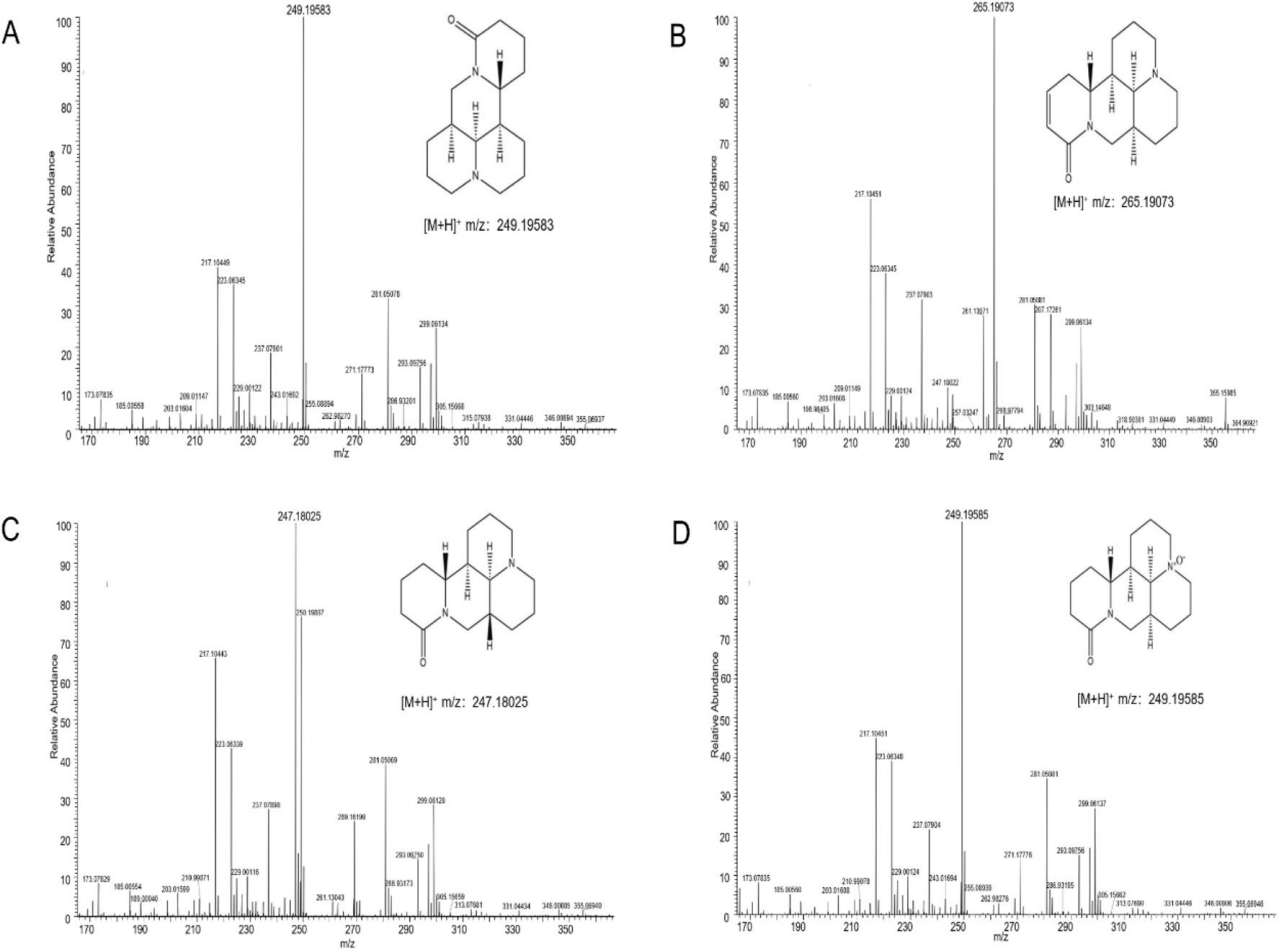
The mass spectrum of the four alkaloids (**A**) Matrine, (**B**) Sophoridine, (**C**) Oxymatrine, (**D**) Sophocarpine.

The retention times of mebendazole (m/z 296.1030), matrine (m/z 249.1959), sophoridine (m/z 249.1959), oxymatrine (m/z 265.1907) and sophocarpine (m/z 247.1803) were 8.93, 2.65, 3.86, 3.64, and 2.92 min, respectively (see Figs. 4 and 5). The method has good separation and no interference from endogenous substances.

#### Linearity and LLOQ

The linear ranges of matrine, oxymatrine, sophocarpine and sophoridine were 0.80–206.00, 0.87–224.00, 0.73–188.00 and 0.75–192.00 ng/mL, respectively. The linear relationship of the four alkaloids in biological samples was good, R^2^ > 0.99, and the linear model was suitable. The standard curve is shown in Table 6. The LLOQs values of the four alkaloids were all 1 ng/mL (S/N > 10), which was sufficient for the quantitative study of the four alkaloids in the pharmacokinetics of low-dose drugs.

**Table 6:** Standard curve table of UPLC-MS method for TAs-SM.

Name	Standard curve	R^2^	Linear range (ng/mL)
Matrine	Y = 0.0124X + 0.0590	0.9912	0.80–206.00
Oxymatrine	Y = 0.0065X + 0.0225	0.9916	0.87–224.00
Sophocarpine	Y = 0.0072X + 0.0216	0.9928	0.73–188.00
Sophoridine	Y = 0.0124X + 0.0507	0.9915	0.75–192.00

#### Accuracy and precision

The intra-day and inter-day accuracy ranges of the four alkaloids were 0.64–7.99% and 0.64–7.60%, respectively; The intra-day and inter-day precision ranges were 0.65–6.08% and 0.51–7.65%, respectively. All of these values are less than 10%, meeting the requirements of accuracy and precision^28^. The results are shown in Table 7.

**Table 7:** Inter- and intra-day accuracy and precision for the determination of total alkaloid in rat plasma (n = 5).

Name	Spiked concentration (ng/mL)	Inter-day			Intra-day		
		Concentration (mean ± SD)	Precision (%)	Accuracy (%)	Concentration (mean ± SD)	Precision (%)	Accuracy (%)
Matrine	1.56	1.58 ± 0.02	1.27	1.28	1.57 ± 0.03	1.91	0.64
	25.00	26.92 ± 1.39	5.16	7.68	26.25 ± 0.89	3.39	5.00
	100.00	107.63 ± 4.87	4.52	7.63	104.70 ± 1.56	1.49	4.70
Oxymatrine	1.56	1.57 ± 0.04	2.55	0.64	1.66 ± 0.05	3.01	6.41
	25.00	26.58 ± 0.81	3.05	6.32	26.90 ± 0.22	0.82	7.60
	100.00	107.26 ± 3.54	3.30	7.26	106.88 ± 0.85	0.80	6.88
Sophocarpine	1.56	1.55 ± 0.04	2.58	6.41	1.54 ± 0.02	1.30	1.28
	25.00	24.00 ± 1.46	6.08	4.00	26.68 ± 2.04	7.65	6.72
	100.00	107.99 ± 2.67	2.47	7.99	105.31 ± 0.91	0.86	5.31
Sophoridine	1.56	1.53 ± 0.01	0.65	1.92	1.57 ± 0.03	1.91	0.64
	25.00	26.32 ± 0.99	3.76	5.28	26.37 ± 1.83	6.94	5.48
	100.00	106.23 ± 2.67	2.51	6.23	107.44 ± 0.55	0.51	7.44

#### Extraction recovery and matrix effect

The recoveries of the four alkaloids at low, medium and high concentrations ranged from 0.52 to 5.54%, and the matrix effect ranged from 0.33 to 3.84%, which was in the range of 85–115% of biological samples (Table 8).

**Table 8:** Extraction recovery and Matrix effect for the assay of total alkaloid in rat plasma (n = 5).

Name	Spiked plasma concentration (ng/mL)	Extraction recovery (%, mean ± SD)	RSD (%)	Matrix effect (%,mean ± SD)	RSD (%)
Matrine	1.56	104.99 ± 3.58	3.41	104.23 ± 3.34	3.20
	25.00	104.70 ± 1.56	1.49	103.12 ± 1.26	1.22
	100.00	101.46 ± 0.58	0.54	102.32 ± 0.53	0.52
Oxymatrine	1.56	105.20 ± 5.83	5.54	103.64 ± 3.98	3.84
	25.00	106.88 ± 0.85	0.79	104.85 ± 0.95	0.91
	100.00	100.68 ± 1.60	0.87	99.68 ± 1.34	1.34
Sophocarpine	1.56	107.49 ± 5.19	4.83	103.87 ± 3.45	3.32
	25.00	105.31 ± 0.91	0.87	102.16 ± 0.99	0.97
	100.00	103.86 ± 1.98	1.91	103.78 ± 1.43	1.38
Sophoridine	1.56	104.48 ± 0.95	0.91	105.89 ± 0.87	0.82
	25.00	107.44 ± 0.55	0.52	104.56 ± 0.34	0.33
	100.00	102.59 ± 0.82	0.80	101.86 ± 0.54	0.53

#### Stability

The stability of the four alkaloids at 25 °C for 24 h, 4 °C for 24 h, and − 80 °C for 15d are shown in Table 9. The RSD ranged from 0.63 to 5.92%, which met the requirements of biological sample determination. The results showed that the four alkaloids had good stability.

**Table 9:** The stability for the determination of total alkaloid in rat plasma (n = 5).

Name	Concentration (ng/mL)	Room temperature for 24 h		4 °C for 24 h		Three freeze		At − 80 °C for 15 days	
		Measured (mean ± SD)	RSD (%)	Measured (mean ± SD)	RSD (%)	Measured (mean ± SD)	RSD (%)	Measured (mean ± SD)	RSD (%)
Matrine	1.56	1.56 ± 0.05	3.21	1.60 ± 0.03	1.88	1.59 ± 0.09	5.66	1.58 ± 0.02	1.27
	25.00	26.45 ± 0.65	2.46	25.90 ± 0.80	3.09	25.33 ± 1.50	5.92	24. 94 ± 1.36	5.45
	100.00	104.44 ± 6.74	6.45	113.83 ± 1.52	1.34	102.73 ± 0.96	0.93	101.47 ± 3.70	3.65
Oxymatrine	1.56	1.59 ± 0.05	3.14	1.55 ± 0.02	1.29	1.60 ± 0.01	0.63	1.54 ± 0.04	2.60
	25.00	25.35 ± 1.43	5.64	25.64 ± 0.33	1.29	24.88 ± 0.71	2.85	25.60 ± 1.01	3.95
	100.00	103.26 ± 3.14	3.04	105.91 ± 0.92	0.87	101.26 ± 2.03	2.00	108.91 ± 1.68	1.54
Sophocarpine	1.56	1.55 ± 0.08	5.16	1.53 ± 0.06	3.92	1.60 ± 0.03	1.88	1.54 ± 0.06	3.90
	25.00	25.31 ± 0.76	3.00	25.27 ± 0.21	0.83	24.41 ± 0.85	3.48	24.37 ± 1.39	5.70
	100.00	106.05 ± 1.65	1.56	109.60 ± 0.48	0.44	104.38 ± 3.38	3.24	105.31 ± 2.61	2.48
Sophoridine	1.56	1.55 ± 0.06	3.87	1.57 ± 0.09	5.73	1.62 ± 0.03	1.85	1.63 ± 0.07	4.29
	25.00	24.98 ± 0.33	1.32	24. 12 ± 0.73	3.03	24.64 ± 0.78	3.17	24.91 ± 0.65	2.61
	100.00	102.25 ± 1.17	1.14	109.66 ± 1.37	1.25	101.62 ± 1.14	1.12	108.53 ± 2.82	2.60

### Comparison of systemic pharmacokinetics in SD rats

**Figure 6:**
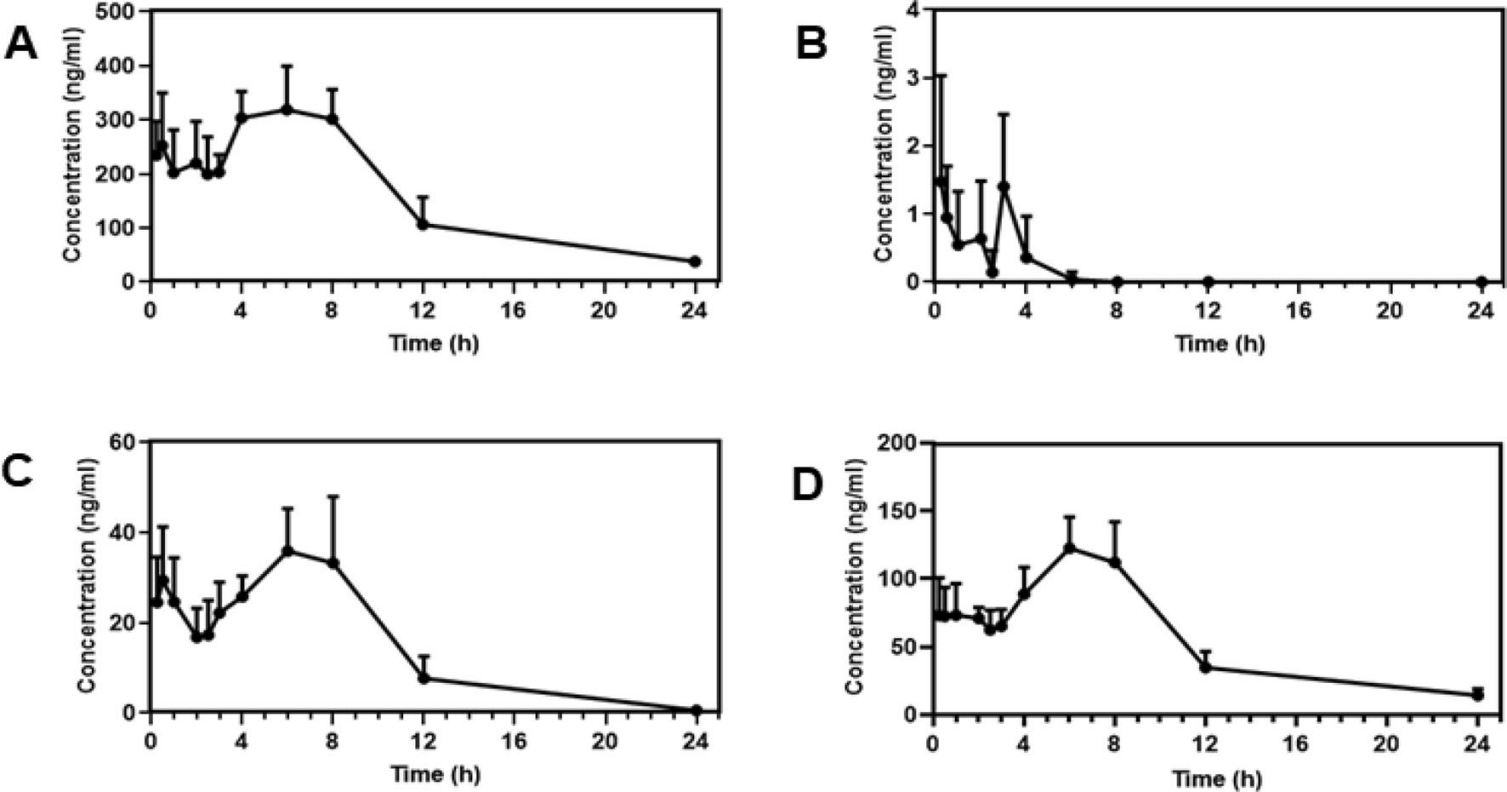
The drug-time curve of the four components of TA-SM. (**A**) Matrine, (**B**) Oxymatrine, (**C**) Sophocarpine, (**D**) Sophoridine.

After the orally-administered TA underwent hepatic metabolism, bimodal phenomenon occurred in all four alkaloids. Matrine first decreased in blood and then peaked at about 6 h with a mean value of 318.65 ng/mL. The in vivo blood concentration of oxymatrine was extremely low, with the highest mean concentration at 3 h, at 1.4 ng/mL; after 6 h, oxymatrine was barely detectable in the blood, which might be related to the low amount of oxymatrine extracted at the time of extraction. The trend for sophorine was the same as matrine, first decreasing and then increasing, with the lowest value reached at 2 h, with an average concentration of 16.81 ng/mL. Then serum concentration began to rise after 2 h and reached a peak value at 6 h with an average concentration of 35.89 ng/mL, and there was essentially no sophorine detected at 24 h. Sophoridine reached the highest peak concentration of 122.62 ng/mL at 6 h. The drug time profiles of the four alkaloids are shown in Fig. 6.

Based on the known pharmacokinetic parameters (Table 10), the four alkaloids differed in their half-life, with the fastest metabolized being sophorine, which had a t_1/2_ = 2.64 ± 1.15 h, and the slowest metabolized being sophoridine which had a t_1/2_ = 6.02 ± 1.38 h. There were also differences in peak concentration (*C*_max_) and peak time (*T*_max_): For oxymatrine (*C*_max_ = 2.28 ± 1.02 ng/mL), *T*_max_ was the fastest (1.39 ± 1.27 h). The *C*_max_ of matrine was the highest (381.20 ± 29.51 ng/mL). There was also a difference in the area under the drug-time curve, with the largest AUC_(0−t)_ for matrine (3797.53 ± 615.89 h/ng/mL) and the smallest for oxymatrine (3.36 ± 1.69 h/ng/mL).

**Table 10:** The non-compartmental PK parameters of TA after oral administration of 3 mg/kg (n = 10).

Parameter	Matrine	Oxymatrine	Sophocarpine	Sophoridine
t_1/2_ (h)	5.33 ± 0.87	3.82 ± 6.61	2.64 ± 1.15	6.02 ± 1.38
V (L/kg)	0.05 ± 0.01	190.29 ± 259.77	0.29 ± 0.17	0.15 ± 0.03
CL (L/h/kg)	0.01 ± 0.00	10.59 ± 8.13	0.07 ± 0.02	0.02 ± 0.00
AUC_(0-t)_ (h/ng/mL)	3797.53 ± 615.89	3.36 ± 1.69	348.96 ± 72.16	1312.38 ± 116.85
*C*_max_ (ng/mL)	381.20 ± 29.50	2.28 ± 1.02	42.19 ± 11.77	134.59 ± 20.39
*T*_max_ (h)	5.50 ± 2.60	1.39 ± 1.27	5.00 ± 3.20	6.00 ± 1.16
AUC_(0-∞)_ (h/ng/mL)	4069.04 ± 606.69	3.37 ± 1.69	352.08 ± 70.48	1442.56 ± 110.42

In this study, UPLC-HR-ESI-MS was used for the first time to establish an in vivo detection method for the four alkaloids extracted from SM. The methodological validation of each index was within the regulation range, indicating that the established detection method was accurate and reliable, and the study of the in vivo pharmacokinetics of total alkaloids in rats was feasible with this method. Meanwhile, the pharmacokinetic examination of the TAs-SM found that the blood concentrations of all four alkaloids exhibited bimodal profiles in normal SD rats. This may be because after entering rats, the TAs-SM obtained by SFE-CO_2_ underwent enterohepatic circulation.

The available methods for multiple blood sampling in a short period of time include tail vein, jugular vein and retro-orbital sampling^37^. In the pre-experiment, it was found that subcutaneous congestion occurred in the tail after blood collection from the tail vein sampling at several sampling points, resulting in the failure of the experiment. Jugular vein sampling needs a special and biocompatible hose. However, due to the COVID-19, we can't purchase such a hose. Finally, we had to choose retro-orbital sampling with relatively poor animal welfare. Retro-orbital sampling is one of the recognized blood collection techniques in rats. Blood collection under anesthesia can ensure that animals can collect blood in a painless state, and eye treatment can be given in time after blood collection. Further for retro-orbital sampling no pre-study preparation like cannulation is required before dosing the animals^38^. It conforms to the 3R principle and the rigorous protocol adopted by the animal ethics committee.

It can be seen from the figure that the *C*_max_ of oxymatrine is (2.28 ± 1.02) ng/mL, which is close to the lower limit of quantification, which is theoretically unconventional. This is due to the fact that oxymatrine is easily reduced to matrine in the body, resulting in a lower content^39,40^. However, in order to clarify the pharmacokinetic behavior of the four alkaloids in the body, we have determined the *C*_max_ of oxymatrine and it was also reported.

Evidence of bimodal profiles is inconsistent with the reported pharmacokinetic behavior of alkaloid monomers^41^. This may reflect the multi-component characteristics of traditional Chinese medicines: most traditional Chinese medicines contain a large group of components with the same parent nucleus. Under the environmental conditions in the body, these components are easy to transform into each other, and the mutual transformation of the components will lead to the concentration of a certain component rising again, resulting in multi-peak phenomenon. In this study, matrine, oxymatrine, sophorine and sophoridine are all quinolizidine alkaloids with the same parent nucleus. Therefore, these alkaloids can easily be transformed into each other in vivo and create bimodal or multi-modal phenomena.

Several limitations to this study exist. Firstly, due to the limited experimental conditions in animal study, researchers chose retro-orbital sampling instead of intubation blood collection, which has better ethics. In the future, researchers will pay more attention to the ethical of animal study. Secondly, the supercritical extraction method used in this study is only suitable for small-scale preparation. There is still a lot of work to be done in the future on how to convert small-scale preparation into large-scale production. Finally, in the pharmacokinetic, the drug concentration–time curve of the four alkaloids in rats showed a multi-peak phenomenon. We speculated that this may be related to the related transformation between alkaloids by consulting the literature, but the specific scientific basis needs further research and confirmation.

## Conclusion

In this study, an SFE-CO_2_ method for extracting TAs-SM was established and optimized for the first time. The results of a Box-Behnken response surface analysis indicate that the optimal extraction SFE conditions are as follows: pressure = 31 MPa, temperature = 70 °C, time = 162.18 min; with these parameters the TAs were extracted from each gram of SM, with the total content being 68.88 μg. In order to further analyze the drug metabolism behavior of TAs in vivo, we established for the first time a sensitive quantitative analysis method using UPLC-HR-ESI-MS. This method was successfully applied to the study of the pharmacokinetics of matrine, oxymatrine, sophorine, and sophoridine after an oral administration. The TA pharmacokinetic curves all showed bimodal profiles, which may reflect the multi-component characteristics of traditional Chinese medicine.
